# Development of a Biocontained Toluene-Degrading Bacterium for Environmental Protection

**DOI:** 10.1128/spectrum.00259-21

**Published:** 2021-07-28

**Authors:** Masahito Ishikawa, Takaaki Kojima, Katsutoshi Hori

**Affiliations:** a Department of Biomolecular Engineering, Graduate School of Engineering, Nagoya University, Nagoya, Japan; b PRESTO, Japan Science and Technology Agency, Kawaguchi, Saitama, Japan; c Laboratory of Molecular Biotechnology, Graduate School of Bioagricultural Sciences, Nagoya University, Nagoya, Japan; University of Guelph

**Keywords:** biocontainment, insertion sequence, synthetic biology, MinION

## Abstract

Biocontainment is a safeguard strategy for preventing uncontrolled proliferation of genetically engineered microorganisms (GEMs) in the environment. Biocontained GEMs are designed to survive only in the presence of a specific molecule. The design of a pollutant-degrading and pollutant-dependent GEM prevents its proliferation after cleaning the environment. In this study, we present a biocontained toluene-degrading bacterium based on Acinetobacter sp. Tol 5. The *bamA* gene, which encodes an essential outer membrane protein, was deleted from the chromosome of Tol 5 but complemented with a plasmid carrying a *bamA* gene regulated by the Pu promoter and the regulatory protein XylR. The resultant strain (PuBamA) degraded toluene, similarly to the wild-type Tol 5. Although the cell growth of the PuBamA strain was remarkably inhibited after toluene depletion, escape mutants emerged at a frequency of 1 per 5.3 × 10^−7^ cells. Analyses of escape mutants revealed that insertion sequences (ISs) carrying promoters were inserted between the Pu promoter and the *bamA* gene on the complemented plasmid. MinION deep sequencing of the plasmids extracted from the escape mutants enabled the identification of three types of ISs involved in the emergence of escape mutants, suggesting a strategy for reducing it.

**IMPORTANCE** GEMs are beneficial for various applications, including environmental protection. However, the risks of GEM release into the environment have been debated for a long time. If a pollutant is employed as a specific molecule for a biocontainment system, GEMs capable of degrading pollutants are available for environmental protection. Nevertheless, to our knowledge, biocontained degraders for real pollutants have not been reported in academic journals so far. This is possibly due to the difficulty in the expression of enzymes for degrading pollutants in a tractable bacterium such as Escherichia coli. On the other hand, bacteria with enzymes for degrading pollutants are often intractable as a host of GEMs due to the shortage of tools for genetic manipulation. This study reports the feasibility of a biocontainment strategy for a toluene degrader. Our results provide useful insights into the construction of a GEM biocontainment system for environmental protection.

## INTRODUCTION

Advances in synthetic biology have prompted the use of genetically engineered microorganisms (GEMs) as versatile agents for biomedical, industrial, and environmental applications (1–3). Regarding environmental applications, GEMs can be used for sensing and eliminating environmental pollutants, such as organic xenobiotics, robust polymers, and toxic metals (4–6). However, the risks of GEM release into the environment have been debated because the spread and proliferation of GEMs may adversely affect the ecosystem. Biocontainment is a safeguard strategy to prevent the uncontrolled proliferation of GEMs in the environment (7, 8). Biocontainment strategies can be classified according to the mechanisms of restraint. Active biocontainment strategies employ chemically inducible killing switches, such as toxin-antitoxin genes (9–11) and self-destructing DNA (12). Although such GEMs survive when kill switches are blocked by a chemically induced regulatory protein under the assigned biocontainment condition, they commit suicide when the chemical inducer is removed. Passive biocontainment strategies are designed to produce GEMs that are dependent on externally supplied chemicals by modifying the function of essential genes (13–15). The proliferation of such GEMs is repressed in the absence of a specific chemical.

If a pollutant is employed as an inducer of the regulatory protein of the kill switch or as a supplement for auxotrophic GEMs, both biocontainment approaches allow the application of GEMs capable of degrading pollutants for environmental protection. Biocontained pollutant degraders, which commit suicide in the absence of a pollutant or show pollutant auxotrophy, cannot proliferate after cleaning the polluted environment. Ramos et al. proposed this biocontainment approach for environmental protection (16) and reported some biocontained strains, derived from Pseudomonas putida KT2440, that committed suicide after degrading the model pollutant 3-methylbenzonate (3-MB) (17–19). However, 3-MB is not a pollutant that actually causes severe environmental degradation. To our knowledge, although previous reports have demonstrated the feasibility of biocontainment systems for environmental protection, biocontained degraders for real pollutants have not been reported in academic journals so far.

According to the Pollutant Release and Transfer Register issued by the Japanese Ministry of the Environment, toluene is the most abundant volatile organic compound (VOC) emitted in the environment. Inhalation of toluene can cause symptoms that include fatigue, confusion, weakness, drunken-type behavior, memory loss, nausea, loss of appetite, hearing loss, and color vision loss. Biological elimination of toluene has been reported to be a cost-effective treatment process for off-gas containing low concentrations of toluene (20–22). Acinetobacter sp. Tol 5 was isolated as a toluene-degrading Gram-negative bacterium from a biofiltration unit for such a process (20). The complete genome sequence of Tol 5 (DDBJ/GenBank accession numbers AP024708 and AP024709) indicates that Tol 5 metabolizes toluene via the toluene dioxygenase pathway. In this pathway, toluene is oxidized to toluene *cis*-hydrodiol by toluene dioxygenase (TodC1C2BA multiple enzyme complex); toluene *cis*-hydrodiol is then oxidized to 3-methylcatechol by toluene *cis*-dihydrodiol dehydrogenase (TodD). The cleavage of 3-methylcatechol by 3-methylcatechol 2,3-dioxygenase (TodE) yields 2-hydroxy-6-oxo-2,4-heptadieonate, which is further metabolized to 2-hydoroxypenta-2,4-dienoate and acetate by 2-hydroxy-6-oxo-2,4-heptadieonate hydrase (TodF). These metabolites are finally converted to tricarboxylic acid (TCA) cycle intermediates. Owing to the relaxed substrate specificity of toluene dioxygenase (EC 1.14.12.11), Tol 5 can metabolize not only toluene but also other aromatic compounds, including benzene, xylene, and styrene (20). This metabolic trait of Tol 5 is beneficial for biotechnological and environmental applications. We previously established methods for the genetic manipulation of Tol 5 (23, 24) and constructed a genetically engineered Tol 5 strain for biotechnological applications (25–27).

**FIG 1:**
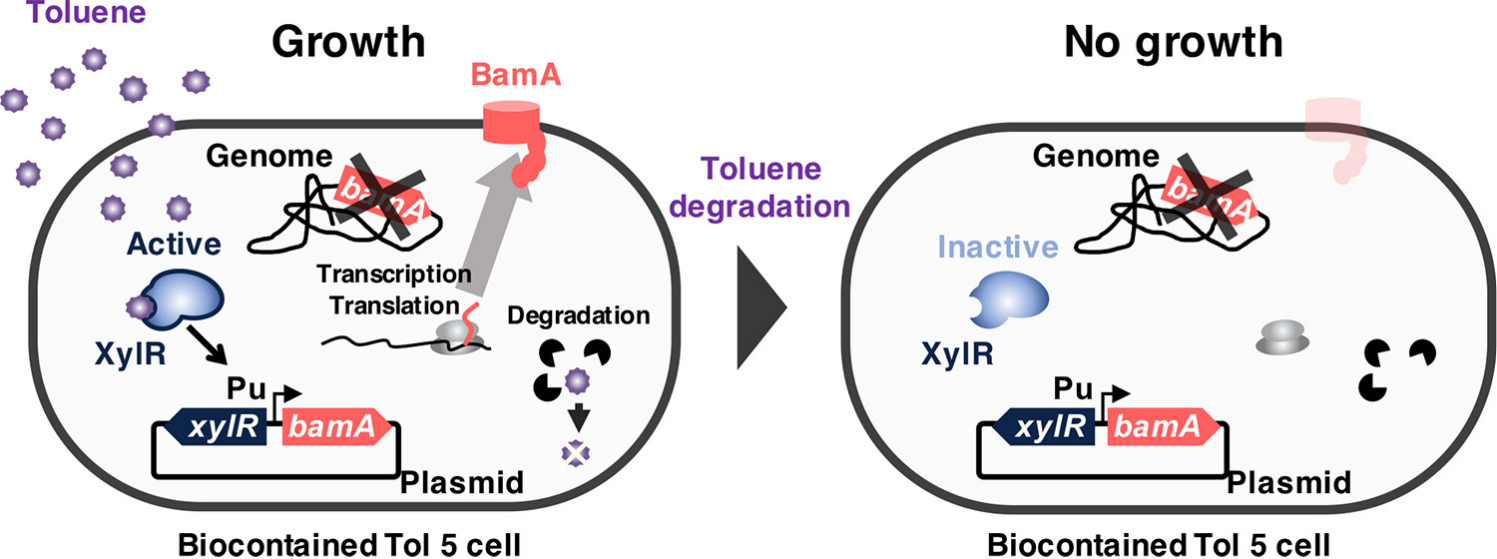
Schematic of the functioning of a biocontained toluene-degrading bacterium. Acinetobacter sp. Tol 5 is a Gram-negative bacterium that metabolizes various carbon sources, including toluene. BamA, a widely conserved outer membrane protein, is essential for cell growth in most Gram-negative bacteria. A *bamA*-deficient mutant of Tol 5 was complemented with a plasmid harboring a *bamA* gene controlled by a XylR-Pu promoter system. XylR is a transcriptional regulatory protein-binding toluene. Toluene-bound XylR activates the Pu promoter. In the presence of toluene, the biocontained Tol 5 can grow due to the transcription and translation of the *bamA* gene from the plasmid. As XylR switches to an inactive form after the complete degradation of toluene, the transcription and translation of the *bamA* gene stop. As a result, the cell growth of biocontained Tol 5 ceases due to the depletion of newly synthesized BamA.

This study aimed to develop a biocontained Tol 5 strain capable of eliminating toluene from polluted environments. The concept of a biocontained toluene degrader is illustrated in Fig. 1. This biocontained strain lacks the *bamA* gene (previously called *yaeT*), which encodes an essential outer membrane protein of Gram-negative bacteria (28), and instead possesses a plasmid carrying a *bamA* gene under the control of the toluene-inducible Pu promoter and its regulator XylR (29). In the presence of toluene, XylR activates the Pu promoter and thus the transcription of the *bamA* gene. Then, the gradual degradation of toluene by this biocontained strain leads to the cessation of *bamA* transcription. After the complete degradation of toluene, this biocontained strain can no longer grow because of the lack of newly synthesized BamA. In this study, we demonstrated the feasibility of this concept.

## RESULTS

### Conditional expression of the *bamA* gene in Acinetobacter sp. Tol 5.

**FIG 2:**
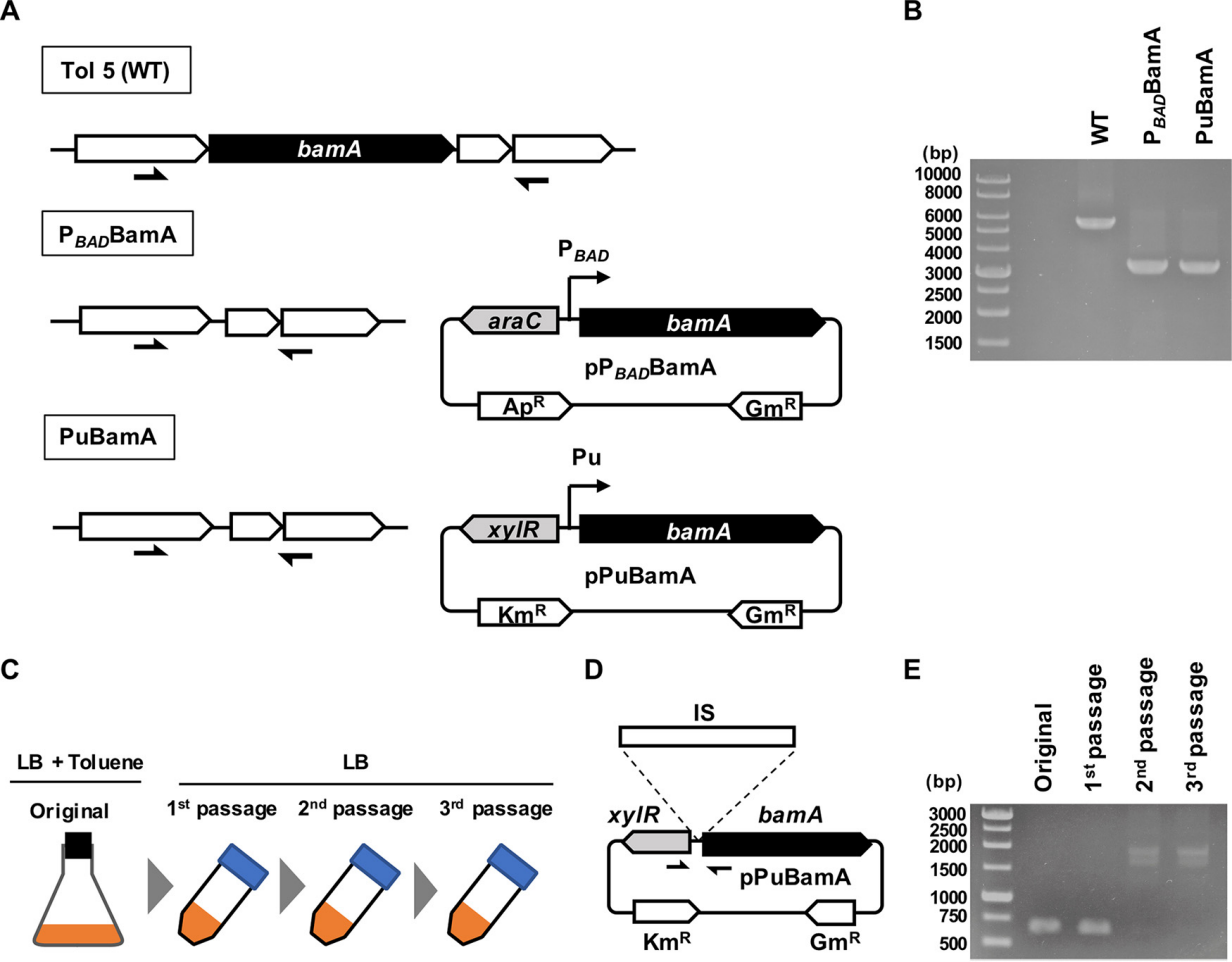
Construction of Acinetobacter sp. Tol 5 mutants expressing the *bamA* gene in a specific condition and analysis of mutants escaping from the biological containment system. (A) Schematic diagrams representing the genomic organization around the *bamA* gene in the chromosome of Tol 5 and the plasmid for complementation. Half-arrowheads indicate the locations where primers anneal. (B) PCR confirmation of *bamA* disruption. PCR was performed using the primers BamA_p1_F and BamA_p2_R. The target sizes were 5,701 bp (wild type [WT]) and 3,392 bp (*bamA*-deficient mutants). (C) Schematic of the procedure to enrich cultures for mutants escaping from the biocontainment system. The PuBamA strain was serially passaged in LB medium not supplemented with toluene. (D) Schematic of insertion sequence (IS) insertion into the Pu promoter on the pPuBamA plasmid. Half-arrowheads indicate the positions where the primers IS-P-ChecK_F and IS-P-Check_R anneal. PCR using these primers amplifies a 663-bp DNA fragment in the absence of an IS element. (E) PCR amplification of the DNA region around the Pu promoter.

**FIG 3:**
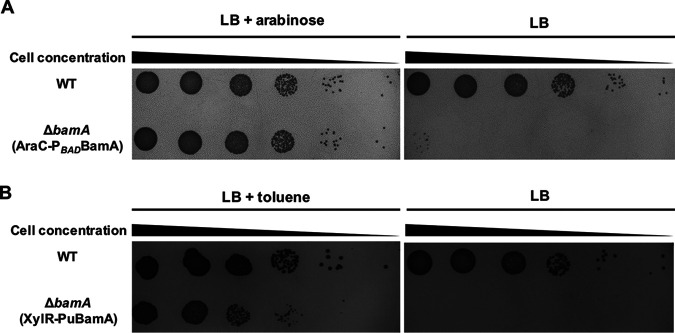
Cell growth of Acinetobacter sp. Tol 5 strains with conditional expression of *bamA* on agar plates. (A) Growth of a *bamA*-deficient mutant carrying the *bamA* gene under the control of an arabinose-inducible promoter. (B) Growth of a *bamA*-deficient mutant carrying the *bamA* gene under the control of a toluene-inducible promoter. Cell suspensions of the wild-type (WT) and the *bamA* conditional expression mutant strains were serially diluted 1:10. Each serial dilution was spotted onto LB agar plates.

To examine whether the *bamA* gene is essential for the cell growth of Tol 5, similarly to other Gram-negative bacteria, we attempted to delete the *bamA* gene from the chromosome of Tol 5 through a typical plasmid-based method using the *sacB* gene as a counterselectable marker (23) (see Table 1 for strains and plasmids used in this study). Single-crossover mutants, in which the suicide plasmid was integrated in proximity to the *bamA* gene, were successfully constructed. However, no double-crossover mutant lacking the *bamA* gene was obtained (data not shown). After the transformation of a single-crossover mutant with a plasmid carrying the *bamA* gene under the control of an arabinose-inducible promoter (P*_BAD_*), we successfully retrieved a double-crossover Tol 5 mutant lacking the *bamA* gene on the chromosome, the P*_BAD_*BamA mutant, from an agar plate containing 5% sucrose and 0.5% arabinose. The deletion of the *bamA* gene was confirmed by PCR using primers annealing to the sequences upstream and downstream of the region flanking the *bamA* gene in Tol 5 (Fig. 2) (see Table 2 for primers used in this study). The PCR amplicon from the P*_BAD_*BamA mutant was shorter than that from the wild-type (WT) Tol 5 strain, indicating successful deletion of the *bamA* gene. The P*_BAD_*BamA mutant was grown in liquid medium containing 0.5% arabinose and then spotted on LB agar plates containing either no or 0.5% arabinose (Fig. 3A). The WT grew on both plates, whereas the P*_BAD_*BamA mutant hardly grew on the LB plate containing no arabinose, indicating that the *bamA* gene is essential for the cell growth of Tol 5, similarly to other Gram-negative bacteria.

**TABLE 1:** Bacterial strains and plasmids used in this study

Species, strain, or plasmid	Description^*a*^	Source or reference
Acinetobacter sp. Tol 5		
WT	Wild-type strain	20
SC-*bamA*KO	Single-crossover mutant in which the pJQBamAKO plasmid was integrated in the proximity of the *bamA* gene	This study
SC-*bamA*KO (pP*_BAD_*BamA)	SC-*bamA*KO transformed with the pP*_BAD_*BamA plasmid	This study
P*_BAD_*BamA	Δ*bamA* mutant complemented with pP*_BAD_*BamA	This study
PuBamA	Δ*bamA* mutant complemented with pPuBamA	This study
Escherichia coli		
XL10-Gold	Host for routine cloning	Agilent
S17-1	Donor strain for bacterial conjugation	45
Pseudomonas putida		
mt-2	Source of *xylR* and the Pu promoter	46
Plasmids		
pJQ200sk	Suicide plasmid, Gm^r^, SacB	47
pJQBamAKO	A DNA fragment containing the upstream and downstream regions of *bamA* ligated into the BamHI site of pJQ200sk	This study
pARP3	E. coli-Acinetobacter shuttle expression vector, *araC-*P*_BAD_*, Amp^r^, Gm^r^	23
pARKM	Substitution of AmpR with Km^r^ on pARP3, *araC-*P*_BAD_*, Km^r^, Gm^r^	48
pP*_BAD_*BamA	pARP3 harboring *bamA*	This study
pPuBamA	Substitution of the region of *araC*-P*_BAD_* with a DNA fragment containing *xylR*, the Pu promoter, and *bamA* on pARPKM, *XylR*–Pu, Km^r^, Gm^r^	This study

a Gm^r^, gentamicin resistance; Amp^r^, ampicillin resistance; Km^r^, kanamycin resistance.

**TABLE 2:** Primers used in this study

Primer	Sequence (5′→3′)
BamA_F	CCCGTTTTTTTGGGCTAGCGAATTCTTACTGGAAAATAATAGGCATGCAGC
BamA_R	CCACCGCGGTGGCGGCCGCTCTTTAAATATTAAAGAAAGATCGTACTTAG
BamA_p1_F	CAGACAGATCAACTTGCCCGC
BamA_p1_R	CCTAAGCCCATCACCATCATTTTCATTG
BamA_p2_F	GCGGACATTTGGTTTACTACATTATTGAAGC
BamA_p2_R	AGTTACACGATCTACAAGCAAGAACGG
BamA_1500_F	CGAATTCCTGCAGCCCGGGGCGCTGATCATGAGTGTAC
BamA_1500_R	TCCAATCATGGAATATCACGTGCAATGAAC
BamA_1700_F	CGTGATATTCCATGATTGGACCACTTTC
BamA_1700_R	CGGCCGCTCTAGAACTAGTGGTAGAGATTTAGACTCAGTC
PU_F	TCGTCGCCGCGTTTAAACGGGAAAGCGCGATGAACC
PU_R	TGCCTATTATTTTCCAGTAAGAATTCGACTCCAGGCGTAACGGC
IS-P-ChecK_F	TCTTGGGTTTGTATGTAAGCG
IS-P-ChecK_R	TTGCTGTACAGCTGCCATTG
XylR_F	AATTGGGTACCGGGCCCCCCCTCGAGTTTTCACACAACCTGGGGCG
XylR_R	CGCTTTCCCGTTTAAACGCGGCGACGAATTGGCGG

To obtain a Tol 5 mutant dependent on toluene for cell growth, we constructed a pPuBamA plasmid in which *bamA* gene expression was controlled by toluene. Although the plasmid backbone of pPuBamA is the same as that of pP*_BAD_*BamA, pPuBamA harbors *xylR*, a Pu promoter, and a kanamycin resistance gene (Km^r^) instead of *araC*, a P*_BAD_* promoter, and an ampicillin resistance gene (Ap^r^). XylR, a regulatory protein found in Pseudomonas putida mt-2, which activates the Pu promoter only when bound to toluene. Hence, *bamA* gene expression was under the control of a toluene-inducible system in the constructed pPuBamA plasmid, which was used to transform the P*_BAD_*BamA strain. Plasmid incompatibility enabled replacement of the pP*_BAD_*BamA plasmid with the pPuBamA plasmid in the P*_BAD_*BamA strain. The resulting transformant, the PuBamA strain, was examined for its dependency on toluene by plating tests (Fig. 3B). While the WT strain formed colonies on LB plates regardless of toluene supplementation, the PuBamA strain only grew on LB plates supplemented with toluene vapor, indicating its toluene auxotrophy.

### Toluene degradation and biocontainment in toluene-polluted medium.

**FIG 4:**
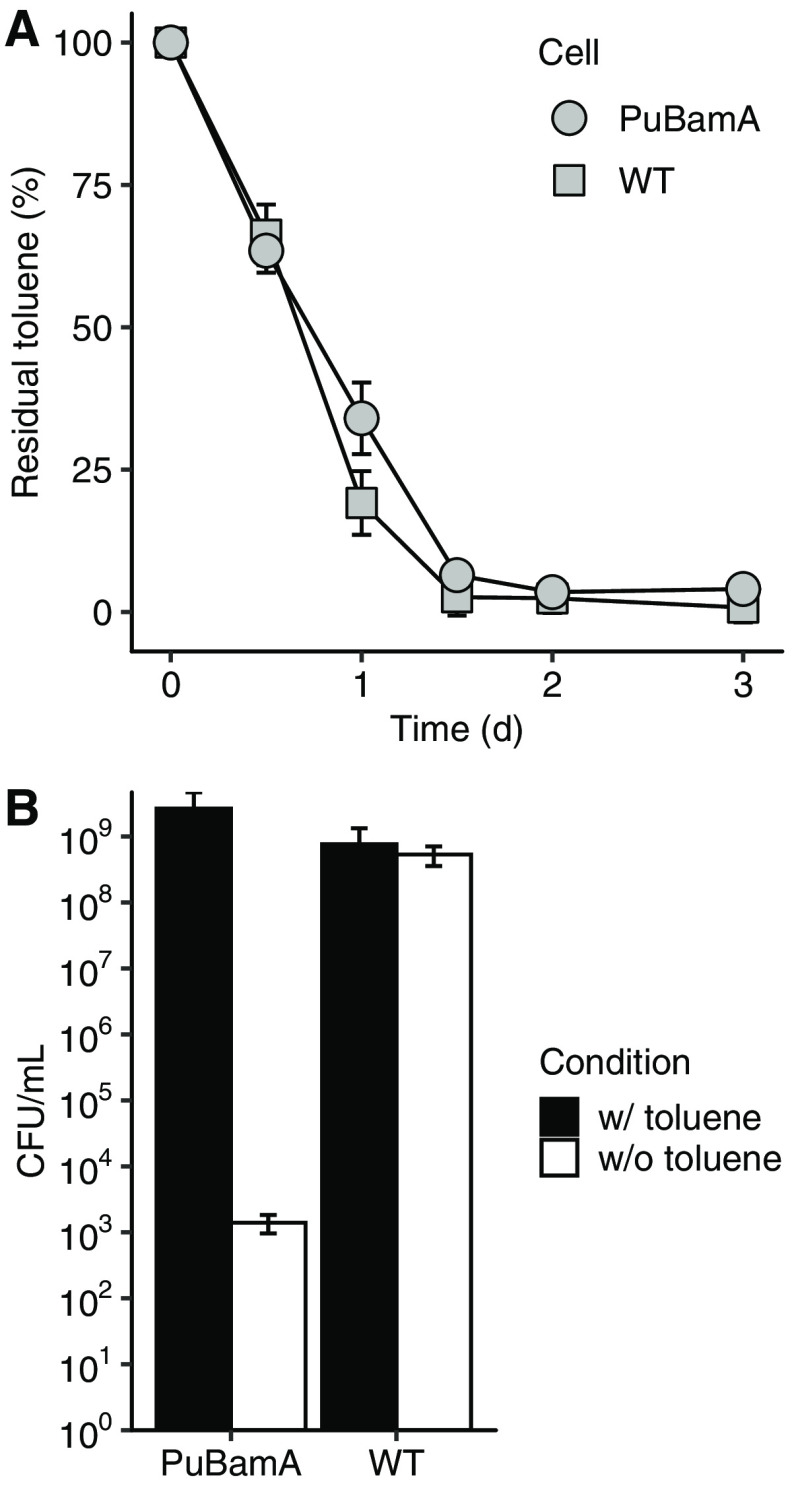
Biocontainment of a toluene-degrading bacterium. (A) Toluene degradation by the wild type (WT) and by a Δ*bamA* mutant carrying the *bamA* gene under the control of a toluene-inducible system (PuBamA). Data are presented as mean ± standard deviation (*n* = 3). (B) Bacterial viability after toluene degradation. After a 3-day incubation of the toluene degradation reaction shown in panel A, cell suspensions of the WT and PuBamA strains were spotted on LB agar plates supplemented or not supplemented with toluene vapor for a CFU test. Data are presented as mean ± standard error (*n* = 3).

To evaluate our biocontainment system for introduction into the environment for bioremediation purposes, the PuBamA strain was examined for its ability to degrade toluene and its viability after toluene degradation. Approximately 8.4 × 10^8^ WT and PuBamA cells were inoculated into liquid LB medium supplemented with 0.05% (vol/vol) toluene. Figure 4A shows the time course of toluene concentration in the medium. Both strains degraded almost all toluene within 3 days, revealing that the PuBamA strain was able to degrade toluene at the same level as the WT strain. After a 3-day incubation, each cell suspension was placed on LB agar plates for a CFU test (Fig. 4B). Although both strains formed colonies (PuBamA, 2.7 ± 2.0 × 10^9^ CFU/ml; WT, 9.9 ± 5.7 × 10^8^ CFU/ml) on LB agar plates supplemented with toluene vapor (w/toluene), the PuBamA strain formed comparatively smaller colonies (1.4 ± 0.4 × 10^3^ CFU/ml) on LB agar plates without toluene (w/o toluene) than those of the WT strain (5.3 ± 1.8 × 10^8^ CFU/ml). Colonies of the PuBamA strain on the LB agar plate without toluene (w/o toluene) could have been derived from mutants escaping from our biocontainment system. The frequency of emergence of escape mutants was calculated to be 1 per 5.3 ± 1.6 × 10^−7^ cells.

To examine how these escape mutants emerged, we extracted pPuBamA plasmids from five randomly selected escape mutants. Sequencing of these plasmids revealed that the same insertion sequence (IS) was inserted between the Pu promoter and the start codon of the *bamA* gene at two different positions (see Fig. S1 in the supplemental material). A sequence similarity search using ISfinder (http://www-is.biotoul.fr), a database for bacterial ISs (30), allowed us to identify the inserted IS as IS*Abe18*, which is found in some Acinetobacter species, such as Acinetobacter lwoffii and Acinetobacter bereziniae (31). Although the biological functions of IS*Abe18* remain unclear, the growth of mutants with IS*Abe18* transposition in the absence of toluene suggests that IS*Abe18* includes promoters that activate its flaking genes. The putative promoter sequences within IS*Abe18*, predicted by BPROM, a program for the recognition of bacterial σ^70^ promoters (http://www.softberry.com/berry.phtml?topic=bprom&group=programs&subgroup=gfindb), are shown in Fig. S2 in the supplemental material. These promoters may drive transcription of the *bamA* gene independent of toluene, resulting in the survival of the escape mutants in the absence of toluene.

### Targeted deep sequencing of plasmids extracted from escape mutants.

**FIG 5:**
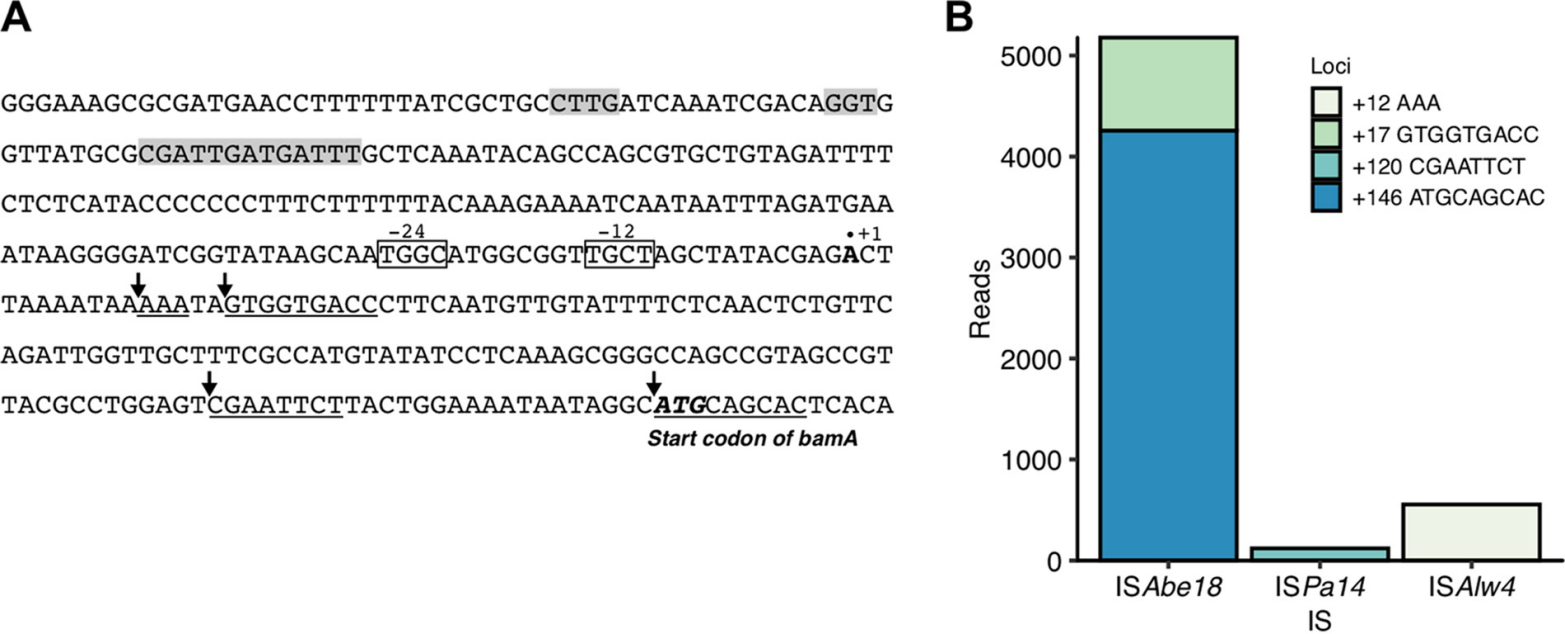
Types of inserted ISs and their insertion sites in the PU promoter. (A) Nucleotide sequence of the region surrounding the Pu promoter in the pPuBamA plasmid. Gray backgrounds, boxes, the dot, underlines, and arrowheads indicate the upstream regulatory sequences, the promoter sequence (−24 and −12), the transcription start site (+1), the direct repeats created by IS elements, and the positions where IS elements were inserted (+12, +17, +120, and +146), respectively. The upstream regulatory sequences, the promoter sequence, and transcription start site correspond to those found by Inouye et al. (44). (B) Distribution of the IS insertion sites. Deep sequencing of PCR amplicons (lane of the third passage in Fig. 2E) revealed that three types of ISs were inserted at different sites in the Pu promoter.

The bacterial genome generally includes multiple IS elements. To examine how many types of IS elements could trigger the emergence of mutants escaping from our biocontainment system, pPuBamA plasmids were extracted from a mixed culture of escape mutants. The escape mutants were enriched in the culture by three serial passages, as shown in Fig. 2C. The enrichment of the escape mutants was confirmed by PCR using the extracted plasmids as a DNA template and primers annealing to the upstream and downstream regions of the *bamA* gene (Fig. 2D). In the absence of an IS element, a 663-bp DNA fragment was amplified by PCR. Larger amplicons, of approximately 1,600 to 1,900 bp, were detected in the second- and third-passage cultures, but not in the original and first-passage cultures, indicating the integration of approximately 900- to 1,200-bp IS elements (Fig. 2E). High-throughput sequencers such as the Illumina MiSeq and Oxford Nanopore Technologies MinION instruments allow for the massively parallel sequencing of a mixture of PCR products. Given the lengths of these amplicons, deep sequencing for the comprehensive identification of IS types and their insertion positions required reads longer than 1,000 bp. Thus, we analyzed the amplicons from the third-passage culture using the MinION nanopore sequencer, which can provide longer reads than Illumina sequencers. Deep sequencing of the amplicons yielded 84,798 reads. The minimum, maximum, and average read lengths were 1 bp, 11,652 bp, and 1,252.6 bp, respectively. The reads were filtered for a minimum length of 600 bp in order to select reads that included IS elements. Clustering analysis based on sequence similarity allowed identification of three types of IS elements (IS*Abe18*, IS*Pa14*, and IS*Alw4*) and four insertion sites (Fig. 5). Information about each IS element is presented in Table 3. These IS elements were found in multiple copies in the complete genome sequence of Tol 5. The BPROM program predicted the presence of putative promoters in IS*Pa14*, IS*Alw4*, and IS*Abe18* (Fig. S2). Approximately 88.5% of the filtered reads included IS*Abe18*, suggesting that IS*Abe18* was the main IS causing the emergence of escape mutants. By examining the flanking direct repeats created by IS elements, the insertion sites of IS*Abe18* were localized at two different positions, +17 and +146, whereas those of IS*Alw4* and IS*Pa14* were observed at positions +12 and +120, respectively. IS*Alw4* and IS*Pa14* were inserted into their sites at the same orientation as shown in Fig. S2, whereas IS*Abe18* was inserted into their sites at both orientations. It is of note that the +146 position lies within the starting codon of the *bamA* gene; this suggests that IS*Abe18* also includes ribosome-binding sites.

**TABLE 3:** IS elements detected by the targeted deep sequencing of escape mutants

Designation	IS family	Length (bp)	Copy number^*a*^	Identity (ISfinder) (no./total [%])
IS*Abe18*	IS*4*	1,269	6	1,262/1,269 (99)
IS*Pa14*	IS*1*	788	3	781/788 (99)
IS*Alw4*	IS*3*	1,309	3	1,309/1,309 (100)

a Copy numbers of the IS elements in the genome of Acinetobacter sp. Tol 5.

## DISCUSSION

This study reports the feasibility of a biocontainment strategy for a toluene degrader. Our biocontainment system incurred the emergence of escape mutants at a frequency of 1 per 5.3 ± 1.6 × 10^−7^ cells, which was slightly higher than the NIH standard (http://osp.od.nih.gov/office-biotechnology-activities/biosafety/nih-guidelines) (one escape mutant per 10^8^ cells). To meet the NIH standard, our biocontainment system requires further improvement. Combination with other biocontainment systems is a promising solution. For example, Ronchel and Ramos successfully decreased the escape frequency of biocontained bacteria by 10-fold by combining a kill switch system with a system regulating an essential gene (17). Therefore, we believe that by adding a kill switch system such as a toxin-antitoxin system to our biocontainment system, we could manage to develop a biocontained toluene degrader that meets the NIH standard. Deletion of IS*Abe18*, IS*Alw4*, and IS*Pa14* from the genome of Tol 5 might also be effective for improving our biocontainment system, because these ISs were responsible for the emergence of the escape mutants. Although their complete deletion is expected to be difficult due to the presence of multiple copies (Table 3), we consider their simultaneous deletion possible if bacterial genome editing technologies can be applied to the Tol 5 strain. In addition, the integration of a gene cassette containing *xylR*, the Pu promoter, and *bamA* into the Tol 5 chromosome is assumed to be an effective approach to reduce the emergence of escape mutants. Bacterial genome editing technologies applicable to the Tol 5 strain would also allow for such genetic manipulation.

In this study, we performed MinION deep sequencing to clarify why escape mutants emerged from our biocontainment system. ISs are the smallest and most numerous transposable elements in bacterial genomes (32). Their length (approximately 0.6 to 2.0 kb) and repeatability have hampered comprehensive analyses by next-generation sequencing based on short-read technologies. In contrast, MinION allows for the deep sequencing of long reads at a low cost. We successfully identified three ISs that caused the emergence of escape mutants from our biocontainment system. Unlike transposons, ISs do not include genes other than those regulating their transposition. Nevertheless, the results of this study suggest that IS*Abe18*, IS*Alw4*, and IS*Pa14* carry promoters and thus play a role in the activation of neighboring genes. Chan et al. reported that an Escherichia coli mutant massively lacking mobile DNA showed a lower escape frequency, suggesting its usefulness as a host strain in biocontainment systems (9). However, E. coli is not always suitable as a host strain in synthetic biology because it cannot functionally express useful enzymes such as the toluene oxygenase. On the other hand, it generally takes a lot of time and resources to construct strains massively lacking mobile DNA in nonmodel microorganisms, such as toluene degraders. For nonmodel microorganisms, it is desirable to minimize the number of mobile DNAs to be deleted. Therefore, we believe that targeting transposable DNA for deletion after demonstrating its role in the emergence of escape mutants by MinION deep sequencing could be an effective approach.

This study employed the *bamA* gene for a passive biocontainment strategy, proposing a new approach. Passive biocontainment strategies generally imply the modification of enzymes involved in metabolism to construct autotrophic GEMs. In contrast, BamA is an outer membrane protein of Gram-negative bacteria that catalyzes the folding and insertion of newly synthesized β-barrel proteins into the outer membrane (33). Since Gram-negative bacteria with a loss of BamA function cannot grow, BamA has been recently identified as a target for the development of new drugs (33–35). We believe that the use of BamA for a passive biocontainment strategy has the following three advantages. (i) BamA is widely conserved in Gram-negative bacteria. Hence, we can engineer many species of Gram-negative bacteria for biocontainment systems if their genome information is available. (ii) BamA is encoded by a single-copy gene. Hence, the modification of only one *bamA* gene is required for biocontainment. This study successfully constructed a biocontained Tol 5 strain with a relatively low escape frequency by single-gene regulation. (iii) BamA has high species specificity (36). Although BamA is widely conserved in Gram-negative bacteria, it only functions in the original species. This feature may be useful for the development of a robust biocontainment system resistant to horizonal gene transfer. Therefore, if a biocontained strain acquired a *bamA* gene from another species of Gram-negative bacteria, it would not function as a factor in the emergence of escape mutants.

The *bamA* gene is under the control of XylR in our biocontainment strategy. Although XylR also activates the Pu promoter in the presence of aromatic compounds other than toluene (37), Tol 5 and its derivative strains can metabolize these compounds through toluene dioxygenase (EC 1.14.12.11). Therefore, if there are aromatic compounds that react with XylR in the environment, they will not cause the escape of a biocontained Tol 5 strain. On the contrary, a biocontained Tol 5 strain could be used for eliminating a variety of aromatic compounds from the environment.

## MATERIALS AND METHODS

### Bacterial culture.

Tol 5 and its derivative strains were grown at 28°C with shaking in a basal salt (BS) medium supplemented with 0.05% (vol/vol) toluene or in LB medium. Escherichia coli strains were grown in LB medium containing the appropriate antibiotics at 37°C. Antibiotics were used at the following concentrations: ampicillin, 500 μg/ml; gentamicin, 10 to 100 μg/ml; and kanamycin, 50 μg/ml for Tol 5-derivative strains; and ampicillin, 100 μg/ml; gentamicin, 10 μg/ml; and kanamycin, 50 μg/ml for E. coli. Arabinose was added to a final concentration of 0.5% (wt/vol) for the induction of *bamA* under the control of an arabinose-inducible promoter. P. putida mt-2 was grown in nutrient broth at 30°C.

### Plasmid construction.

Two E. coli-Acinetobacter shuttle plasmids, pARP3 and pARKM, were used to generate plasmids to complement the *bamA* gene. A DNA fragment containing the *bamA* gene and its ribosome-binding site was amplified by PCR using the primers BamA_F/BamA_R and the genomic DNA of Tol 5 as a template, followed by cloning into the EcoRI-XbaI site of pARP3 using the NEBuilder HiFi DNA assembly system (New England Biolabs, Ipswich, MA) to generate pP*_BAD_*BamA. To obtain a *xylR*-Pu promoter cassette, PCR was performed using the primers XylR_F/XylR_R and PU_F/PU_R and the plasmid pWW0 derived from P. putida mt-2 as a DNA template. The amplified fragments and XhoI-EcoRI-digested pP*_BAD_*BamA were assembled using the NEBuilder HiFi DNA assembly system to generate pARP3-XylR-PuBamA. A XhoI–XbaI-digested fragment containing *xylR*, the Pu promoter, and *bamA* was cloned at the same site as pARPKM to generate pPuBamA.

To generate pJQBamAKO, a suicide plasmid for the deletion of the *bamA* gene, DNA fragments containing the 1.5-kb upstream and 1.7-kb downstream regions of *bamA* were amplified by PCR using the primers BamA_1500_F/BamA_1500_R and BamA_1700_F/BamA_1700_R. The amplicons and BamHI-digested pJQ200sk were assembled using the NEBuilder HiFi DNA assembly system.

### Construction of *bamA*-complemented mutants in Acinetobacter sp. Tol 5.

To obtain a mutant lacking the *bamA* gene on chromosomal DNA and complement it with a *bamA* gene inserted in a plasmid, Tol 5 was conjugated with E. coli S17-1 harboring pP*_BAD_*BamA on an LB agar plate for 24 h at 28°C. The cells were then collected in 1 ml of 0.85% NaCl solution, plated on BS agar plates containing gentamicin (100 μg/ml), and incubated with toluene vapor for 2 days at 28°C. Chromosomal integration of the plasmid was confirmed by colony PCR using the primer sets BamA_p1_F/BamA_p1_R and BamA_p2_F/BamA_p2_R. The resulting single-crossover mutant, SC-*bamA*KO, was conjugated with E. coli S17-1 harboring pP*_BAD_*BamA, as described previously (23). The resulting transformant, SC-*bamA*KO (pP*_BAD_*BamA), was selected on BS agar plates containing ampicillin (500 μg/ml) and gentamicin (10 μg/ml) supplemented with toluene vapor. For the deletion of the *bamA* gene from the chromosomal DNA, SC-*bamA*KO (pP*_BAD_*BamA) was placed on a BS agar plate containing ampicillin (500 μg/ml), gentamicin (10 μg/ml), and 5% sucrose supplemented with toluene vapor, and incubated at 28°C for 2 d. Deletion of the *bamA* gene was confirmed by PCR using the primer set BamA_p1_F/BamA_p2_R.

### Tests for toluene degradation and viability in the absence of toluene.

Overnight cultures of the WT and PuBamA strains were harvested by centrifugation, washed with LB medium, and diluted into 5 ml of LB medium at an optical density at 660 nm (OD_660_) of 0.02 in a 30-ml cylindrical vial containing a 15-mm stirring bar. After adding toluene (2.5 μl) and capping the vial with a butyl rubber septum, the reaction was started with stirring at 28°C. The toluene concentration was measured as previously described (38). After a 72-h incubation, serial dilutions of the cell cultures were prepared in LB medium. Fifty microliters of each dilution were spotted on two LB agar plates; one was routinely incubated at 28°C, and the other was incubated with the supplementation of toluene vapor at 28°C until colonies appeared. Colonies were counted to determine the CFU. The escape frequency was calculated by dividing the number of CFU on the LB plate supplemented with toluene by the number of CFU on the LB plate without toluene. Plasmids from five escape mutants growing on the LB plate without toluene were extracted using a typical alkaline lysis method, digested with XhoI and EcoRI, and analyzed by 1% agarose gel electrophoresis.

### Deep sequencing of PCR amplicons.

One colony of the PuBamA strain was inoculated into 20 ml of LB medium containing 50 μg/ml kanamycin and supplemented with 10 μl of toluene in a 100-ml flask with a butyl rubber cap (original culture). After a 24-h incubation at 28°C with shaking, 50 μl of the culture was inoculated into 5 ml of LB medium containing 50 μg/ml kanamycin in a 50-ml conical tube (first-passage culture). This passage was repeated twice (second- and third-passage cultures). Plasmid DNA (pPuBamA) was extracted from each culture using a typical alkaline lysis method and used as a PCR template. A DNA fragment containing the Pu promoter of the pPuBamA plasmid was amplified by PCR using the primer set IS-P-Check_F/IS-P-Check_R. The resulting amplicon from the third-passage culture was purified using the Wizard SV gel and PCR clean-up kit (Promega, Madison, WI). A sequencing library was prepared using 268 ng of the purified amplicon and a ligation sequencing kit (catalog no. SQK-LSK109; Oxford Nanopore Technologies, Oxford, UK). MinION sequencing was performed using R9.4.1 flow cells (FLO-MIN106; Oxford Nanopore Technologies). MINKNOW software v3.6.3 (Oxford Nanopore Technologies) was used for data acquisition. Real-time base calling was performed with MinIT (Oxford Nanopore Technologies) and the integrated Guppy v3.2.9 software (Oxford Nanopore Technologies) to produce fast5 and fastq files.

### Bioinformatics.

A total of 84,798 reads were filtered by quality and size (*Q* > 10 and >600 bp) using NanoFilt v2.5.0 (39). The filtered fastq files were then converted to fasta files using SeqKit v0.11.0 (40). To identify the types of IS elements inserted in the PuBamA plasmid, the filtered reads were clustered at a 90% identity level using USERCH v11.0.667 (41). Reads in each cluster were subjected to error correction using CONSENT v2.0 (42) and aligned using MAFFT v7.455 (43). Consensus sequences for each cluster were generated from the alignment files using cons (http://emboss.sourceforge.net/apps/cvs/emboss/apps/cons.html) and subsequently analyzed by nucleotide BLAST search using the ISfinder database (http://www-is.biotoul.fr). Each IS insertion site was determined by examining the flanking direct repeats.
