# Supplementary material for: Development of a Biocontained Toluene-Degrading Bacterium for Environmental Protection
Source: Microbiol Spectr. 2021 Jul 28;9(1):10.1128/spectrum.00259-21. doi: 10.1128/spectrum.00259-21 (PMC8552602; doi:10.1128/spectrum.00259-21)
Supplement: SUPPLEMENTAL FILE 1 — Supplemental material. Download SPECTRUM00259-21_Supp_1_seq9.pdf, PDF file, 0.9 MB [file spectrum00259-21_supp_1_seq9.pdf]

Supplemental Material

Development of a biocontained toluene-degrading bacterium for environmental protection

Masahito Ishikawa,[1,2] Takaaki Kojima,[3] and Katsutoshi Hori[1]*

[1]Department of Biomolecular Engineering, Graduate School of Engineering, Nagoya University, Furo-cho, Chikusa-ku, Nagoya 464-8603, Japan
[2]PRESTO, Japan Science and Technology Agency, 4-1-8 Honcho, Kawaguchi, Saitama 332-0012, Japan
[3]Laboratory of Molecular Biotechnology, Graduate School of Bioagricultural Sciences, Nagoya University, Furo-cho, Chikusa-ku, Nagoya 464-8601, Japan

*Corresponding author
E-mail address
khori@chembio.nagoya-u.ac.jp (K. H.)

Figure S1

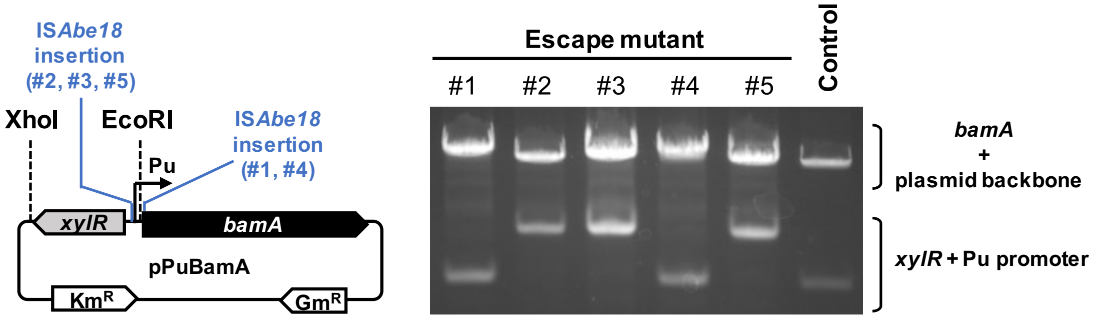

Figure S1. Analysis of plasmids extracted from five escape mutants. The extracted plasmids were digested with XhoI and EcoRI and subsequently analyzed by 1% agarose gel electrophoresis. A pPuBamA plasmid containing no IS was used as a control. The band patterns of the escape mutants were different from those of the control.

>IS*Abe18*

−35 −10

CATTGCTGTCCCACAATTTTTCACAAGAGGAAGCTCATTTGAGCTTCCTCTTGTTTTATGGGTATTTTATCGGGTGAAAATAAAGCTTTTAAAG

TTCTTCTTTCAAACAAATTCACTTGAATTATTCTGAGTAAACGTTGAACTGTCCAACCTGTTTTTCCTAAATGTCGAGCGAAACTCACCAATAA

ATAGGCGATCATCGCAATCCAGATTTGTGTCTGAATTGCGTTCCTGCTGCGGCCTAGAAACGCTTTTAATTTGAGATTCTGCTTAATCGCCTTA

AAGAACAGCTCAACTTTCCAACGATCTTTATAAATCGCCGCAATGGTGGAGGCGGCTAAATGAAAGTTATTGCTGAGAAAGCTAAAGTGCTTGC

−35 −10

CACTTTGCTGATCTCTATATTCAATTCTTCTTAACACTGGGGCTTTTCTTTTTAGGGCATGTGCGCTATTCAGCTGAATGGTTTCATCTTTTAG

AATACCTTTGGATTCAAGCACTGGATGTTGCTGGATCACCTGATACACAGATTTAGGCCTAAAACGTGTGACAAATCCAATGTTTTGAGCAGTC

AGATTTGCATACCATTGGTAATCGACATAGCCTTTATCAAAAACTACAATGCTGCCAGCAGGAAACTGGAATTTGCGGCCTTGTACCATGTCAT

TTTCTTTGCCATTTTCAACTGCAACAAACTCAGGAATATCATTGCTGTGATTCAATCCTATACTGAGTTTCATGCTGGCTTTTGAGTCGTGAAC

TTTGGCCCATTCACATAAAGAAAGCGACAGGTCAATATGACTGGCATCCAAGGAATACAAGGGATTCTTAAAGCGAAATTTATGAGCTACTTTT

−35 −10

GAGTGTTCATAGTATTTAAGCAACTTGTAAAATAGCTGTTGATACAAGGCAGCAGGCTGCTGCTCATTGATTCGTGCCAGCGTGCTTCGGGGAA

TAGACTTTGCTCCGAGATGACTCAGCTTTTCCTGTTGGCACTCCAAATTGGATTGAATATCTCTCAGACTTTGCCTACAAGAGAATTGAGA**CAT**

CAATATGGCAATAAACTGATCCCACCGGGAAGCCGCTCTAAATTTCTGTCCAACATGGTGTACTTTAGCAAGTTGTTCAAAATCCTGTCGCACA

−35

ACAGGTTTAATTAGCTCATGAAATACGGTATTCTGATGTGACAAAACCTGAATCCTGGTCGTTAAAGTGTTTGTTTGCACTCATATTTTAACTG

−10

TTAGGACTCAGGTTTTTTTATTTAAAGCAAACTATGGGACAGCAGTG

>IS*Alw4*

−35 −10

TGAACCGTACCGGGTTTGTCGGAGACCAACTTTCTTGAGAGAATGTCCCA**ATG**ACAAAACCAAAATATACCCCTGAAATCAGAGAAAGAGCGAT

TCAATTATTGATTGAATCTGAAAAAGATTATCCATCGAATTGGGCTGCAATCACAGCAATTGCACCTAAGATTGGCTGTACTCCTGAAACACTA

CGTTCCTGGCATCAGAAGTATTTGAATCAACAGAATCCAATCAAAGTACAGCAGCTTTCAGACCAAGAACGTATCAAACAACTCGAACGCGAAA

ATAAAGAACTGCAACGCGCCAATGAGATTCTACGCAAAGCAGCCGCTTTTTTCGCCCAGGCGGAGCTCGACCGCCCACACAAATA**ATG**GTGGAT

−35 −10

TTTATCCATAATAATAAAGAGCTGTACGGAGTCGAGGCGATTTGTAGAATTTTACCGATTGCAGCTTCGACCTATTATCGGGCTTTAGATCTCG

TTGATAACCCAGAACATCGAGCGAAACGTGCTCTGCATGATTTGCATCATGCAGAGCAAATCAAACGTATTTGGAAAGAAAGTTCAGGTCGATA

TGGTGTACGTAAAGTTTGGCAAAAATTGAAACGTGAGGGTTATGTTATTGCACGTTGTACAGTTGCTCGATTGATGCAAAAGCTAGGTATACAA

GGTGTTTGGCGTGGTAAGAACAAACAAACCACCCGTAGCCGAGATGACCAAAAACGAGCAGATGATTTAGTGAAACGTAATTTTACTGCTGATC

−35 −10

ATCCTGACCAACTGTGGGTGAGTGACTTTACGTATATTCAAACAAATTCAGGCTGGGTTTATACTGCATTTATTATTGATGTGTTCTCACGAGC

AATTGTTGGATGGAAAGTATCTACACGAATGAATACAGATATGGTGCTCGATGCATTAGAGCAAGCATTGCATGATCGAGGCATGCCAAAGAAT

GTGATTCATCATTCCGACAGAGGTGTGCAATATCTTTCCATTCGCTATACCAATCGTTTAGAAGCAGCAAATTTACGAGCATCAGTCGGTACGA

CTGGTGATTCATACGATAATGCTTTGGCTGAAACGGTGAATGGCTTATACAAAACAGAGGTGATTGAATATCTAAAAGCAGATTGGCAAGGTTT

AGCAGATGTACAACTTGCGACACTAAATTGGGTAGATTGGTTCAATAAAGAGCGTGTACATAGTGCACTAGGTTATGTATCACCTTTTGATTTT

−35 −10

GAAGCAATGTACTATGATAAGATTAACCCGTTAGGTCAGGTGGCCTAACTTAAATAAAAAACTCTCCGACAAACCCGGTACGGTTCA

>IS*Pa14*

GGTGGTGTTTCAAAAAGTATGCTGACATTAAATGAAGCCATTATTGATGTAAAAAAAGGTTAAGTCGAAAGCTTTAAAATGGTTTTCAATCTTT

−35 −10

TTTGAAAAATTACAACTCCTTCTAAAACCACGCCTAATTCGATGCCTTATTTTGCAATTATTACCTTCAATACCTACAGTAAAAAATTTACCAA

TACTTTGCTTGCAGTTTTTAAAAGCAGTTATGAAACTGTCCCAATGATCACTTGCAATTCGGGTGTAGTGAATACCTAATTGTTTAAGCTTTGT

CTTCAATCGTTGAACTGTAGCTAAGTCTCTTTTACCCCAAACATAAGCAACAATCTCACCTGTTTCTCGATGGTAGGCGTAAATAAGCCATTGT

−35 −10

TTATTATTTTTATTTCCCACAAAAGTCCAAAACTCATCAACTTCAAGAGACTCATAATGACTTTGTTTAGGCTGAATTTGGTAGGTCGATTCGG

TTAAAGTACGTAAAACTTTACCGATACTGATTCGCTCAACTTCAGCGATATCTCGTATACCACTACCTCTGACCATCAACTGTAATATTTTTCG

AGTAATGCCTGAATTACATCCTAGATAGCTCAGAGCATGGTCACCAATAAACTGACGTTTACAGTCTTTGCACTGATAGTTTTGTTTCCCATCT

−35 −10

ACTTTGATGCCATTTTTCTTTATACTGTCACCGAGGCAGGTTGGACATTTGATTTCTAGAGTTATTCG**CAT**TTCTCTATTTTATCAAAATTCAA

CCTGCTTTGTTTCAGCATACTTTTTGAAACACCACC

Figure S2. Sequences of IS*Abe18*, IS*Alw4*, and IS*Pa14*. Boxes indicate putative promoters predicted by Bprom. Bule- and orange-colored characters indicate sequences coding a transposase. The start codon of each transposase was highlighted by a bold character. Underlines in IS*Abe18* indicate putative ribosome-binding sites.
